# Etymologia: Papillomavirus

**DOI:** 10.3201/eid2005.ET2005

**Published:** 2014-05

**Keywords:** etymologia, papillomavirus, viruses, Richard Shope, Harald zur Hausen, cancer

## Papillomavirus [papʺĭ-loʹmə-viʺrəs]

From the Latin *papillo-* (“nipple”) + *oma* (“tumor”), papillomaviruses are nonenveloped DNA viruses that induce exophytic lesions of the skin and mucous membranes. The first animal papillomavirus was described in 1933 by Richard Shope, who researched papillomata in “warty” wild cottontail rabbits. In 1975, Harald zur Hausen published the hypothesis that the human papillomavirus played a role in the etiology of cervical cancer, work for which he was awarded the Nobel Prize in Physiology or Medicine in 2008.
